# Role of Functional Groups in Tuning Luminescence Signature of Solution-Processed Graphene Quantum Dots: Experimental and Theoretical Insights

**DOI:** 10.3390/molecules29122790

**Published:** 2024-06-12

**Authors:** Zhicheng Ke, Muhammad Azam, Shujat Ali, Muhammad Zubair, Yu Cao, Abbas Ahmad Khan, Ali Hassan, Wei Xue

**Affiliations:** 1 China International Science and Technology Cooperation Base for Laser Processing Robotics, Wenzhou University, Wenzhou 325035, China; 2 National Key Laboratory of Electronic Films and Integrated Devices, School of Integrated Circuit Science and Engineering, University of Electronic Science and Technology of China, Chengdu 610054, China; 3 College of Electrical and Electronic Engineering, Wenzhou University, Wenzhou 325035, China; 4 Forschungszentrum Jülich GmbH, Institute of Energy and Climate Research, Materials Synthesis and Processing (IEK-1), 52428 Jülich, Germany; 5 Oujiang Laboratory (Zhejiang Lab for Regenerative Medicine, Vision and Brain Health), Wenzhou University, Wenzhou 325000, China; 6 IMDEA-Nanociencia, Campus de Cantoblanco, 28049 Madrid, Spain; 7 Zhejiang Provincial Key Laboratory of Laser Processing Robotics, College of Mechanical and Electrical Engineering, Wenzhou University, Wenzhou 325035, China

**Keywords:** graphene, quantum dots, photoluminescence, low-dimensional optoelectronics

## Abstract

Zero-dimensional graphene quantum dots (GQDs) present unique optoelectronic properties in the large-spectrum range from UV to visible. However, the origin of luminescence in GQDs is still a debatable question. Therefore, the present work investigates the features of trap-mediated and edge-state-functionalized group-associated luminescence enhancement of GQDs. The attached functional groups’ involvement in the upsurge of photoluminescence has been discussed theoretically as well as experimentally. In addition, the role of the aromatic ring, the functional group attached, and their positions of attachment to the aromatic ring to tune the emission wavelength and Raman modes have been elucidated theoretically as well as experimentally. We found that in the case of the –OH group attached outside of the aromatic ring, the long-range π hybridization dominates, which suggests that the emission from this model can be dictated by long-range π hybridization. In particular, we found that oxygen-containing functional groups attached outside of the aromatic ring are the main source of the luminescence signature in GQDs. Furthermore, density functional theory (DFT) indicates that the –OH functional group attached outside of the aromatic ring perfectly matched with our experimental results, as the experimental bandgap (2.407 eV) is comparable with the theoretical simulated bandgap (2.399 eV) of the –OH group attached outside of the aromatic ring.

## 1. Introduction

Graphene quantum dots (GQDs), with their nanometric size, have been a matter of great consideration in recent years due to their applications, ranging from optoelectronics to bio-imaging and photodetection in a wide spectral range [1,2,3]. In contrast with two-dimensional graphene, GQDs possess a band gap due to the quantum confinement effect [4,5] and edge effects [6,7] which causes strong photoluminescence in the near band edge region, making them a promising candidate in modern-age nanoscale hybrid sensors [8,9]. The band gap and near-band-edge emission wavelength can be tailored by modulating their size via solution process synthesis [10,11]. Moreover, the charge transfer process in small colloidal semiconductors like quantum dots brings drastic changes, allowing for their application in areas from optoelectronics to bio-imaging, where charges play a significant role [12,13]. The oxygen functionalities of GQDs provide the water solubility which enhances their use in a diverse field of organic luminescent devices. In solution-processed GQDs, the photoluminescence (PL) process (in which electron–hole recombination takes place) strongly depends upon charges [14,15,16,17]. Despite there being a significant role of GQDs in the photoemission process, information on and explanations of photoluminescence features, understanding of the origin and nature of the photoluminescence from these GQDs, and lifetime are still limited. Predictably, PL in GQDs has been ascribed to the quantum confinement effect or surface defects, such as oxygen-containing functional groups (OCFGs) or sp^2^ domains [4,18]. For instance, one popular assumption in this regard suggests that the plausible mechanism for PL in GQDs arises from size-dependent quantum confinements in which the band gap is created between the bonding (π) and antibonding (π*) energy states, and the gap increases with the reduction in the quantum dot size [19,20]. However, other studies have emphasized the significant role of trap-states and functionalized edge-states in controlling the PL behavior, shedding new light on the underlying photophysical mechanisms [21]. It is a consensus that the PL emission of GQDs is a combined effect of aromatic ring structure (i.e., sp^2^ hybridization) and the attached functional groups and defects or a disordered structure (i.e., traps and sp^3^ carbon) [22]. However, the specific role of these functional groups, as well as their attached positions (outside and inside of the aromatic ring structure), has not been investigated systematically. A major hurdle for carrying out such investigation is the production of functionalized GQDs with well-controlled size and similarity in the synthesis process and chemical composition.

Therefore, in the current study we used as-synthesized GQDs to understand the mechanisms of photoluminescence and lifetime decay process influenced by trap-assisted and functionalized edge-states present in solution-processed GQDs. In addition, a comparative analysis of GQDs and graphene has been carried out to check how the PL behavior originated in GQDs. Furthermore, the current study aims to investigate the role of aromatic ring structure, functional groups, and their attached position on the aromatic ring (outside or inside of the aromatic ring) in the photoluminescence signature of solution-processed graphene quantum dots, focusing specifically on the influence of trap states and functionalized edge states. We hypothesize that these structural features play a pivotal role in modulating the emission properties of GQDs, leading to an unprecedented photoluminescence signature. By employing a combination of spectroscopic techniques and theoretical modeling, we seek to elucidate the intricate interplay between trap-states, functionalized edge-states, and photoluminescence behavior in solution-processed GQDs.

## 2. Results

### 2.1. Morphological Characterization

**Figure 1:**
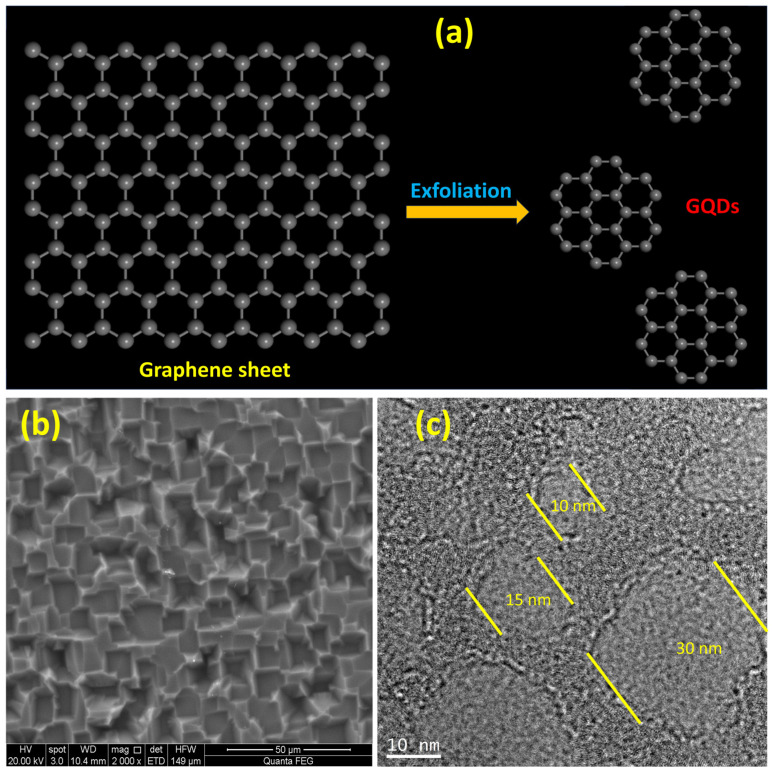
(**a**) Schematic illustration of graphene and graphene quantum dots; (**b**) SEM image of graphene sheet; (**c**) TEM image of graphene quantum dots.

The schematic illustration of GQD formation via graphene sheet exfoliation is presented in Figure 1a. The SEM and TEM images of graphene and graphene quantum dots are presented in Figure 1b and c, respectively. The graphene layer consists of voids with an average area of voids of around 1 micrometer, whereas graphene quantum dots of diameters ranging from ~10–30 nm are present in the TEM image. As in this work, we used a relatively high concentration of graphene quantum dots, without any particular pre-experiment treatment. Nevertheless, the TEM micrograph of GQDs revealed a nearly circular shape. The solution-processed GQDs with almost circular shape exhibited good photoluminescence properties. Moreover, the circular quantum dots additionally had some layers attached at the edges of dots which could be the carbonyl group attached to GQDs, introducing the edge effect. To check the presence of these oxygen functional groups, FTIR spectroscopy was employed, which also confirmed the stretching vibrations from the carbonyl and carboxyl group, as discussed in the next section. 

### 2.2. Fourier-Transform Infrared Spectroscopy

Fourier-transform infrared (FTIR) spectra were analyzed for the characterization of stretching vibrations associated with different functional groups presented in graphene and GQD samples. 

**Figure 2:**
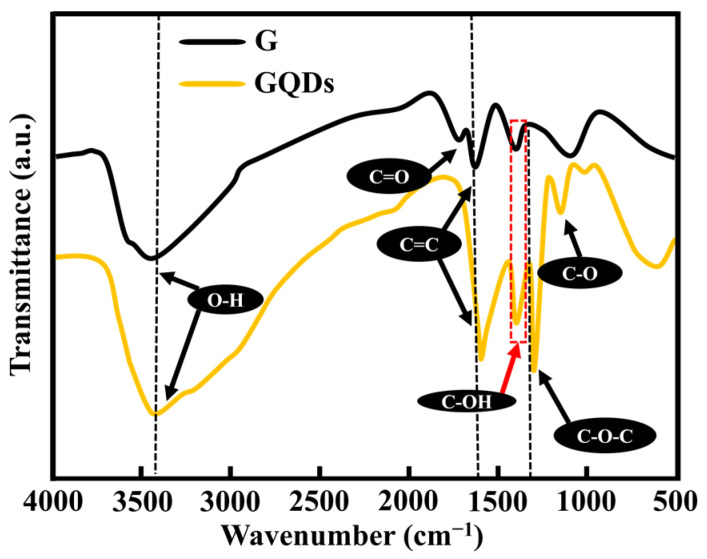
FTIR spectra of GQDs and graphene with stretching vibrations of carbonyl and carboxyl groups.

The FTIR spectra in Figure 2 reveal that the stretching vibrations of hydroxyl groups (O–H) are present in both graphene and GQDs in the broadband spectrum at (3402–3435 cm^−1^). Stretching vibrations of –C=O–, –C=C–, and –C–OH– were observed at 1714 cm^−1^, 1597 cm^−1^, and 1405 cm^−1^, respectively. The observed data are in good agreement with previously reported bands in graphene and GQDs elsewhere [23,24]. 

However, two new stretching vibrational bands have been identified only in GQDs, –C–O–C– at 1250 cm^−1^, and –C–O– at 1043 cm^−1^, respectively. The presence of these stretching vibrations indicates that the oxygen-containing functional groups (OCFGs) are presented at the edges of GQDs, which causes the interlayer distance of GQDs to increase as compared with graphene and graphite [1,25]. Our TEM results in Figure 1c also verify this argument, as the layer at the edges of GQDs can be seen easily. The presence of these OCFGs may tune the optoelectronic properties of GQDs by facilitating solubility and increasing the surface area [26].

### 2.3. Raman Spectroscopy and Component Analysis

The study of the Raman spectrum is very useful for understanding disordered carbon materials [27,28].

**Figure 3:**
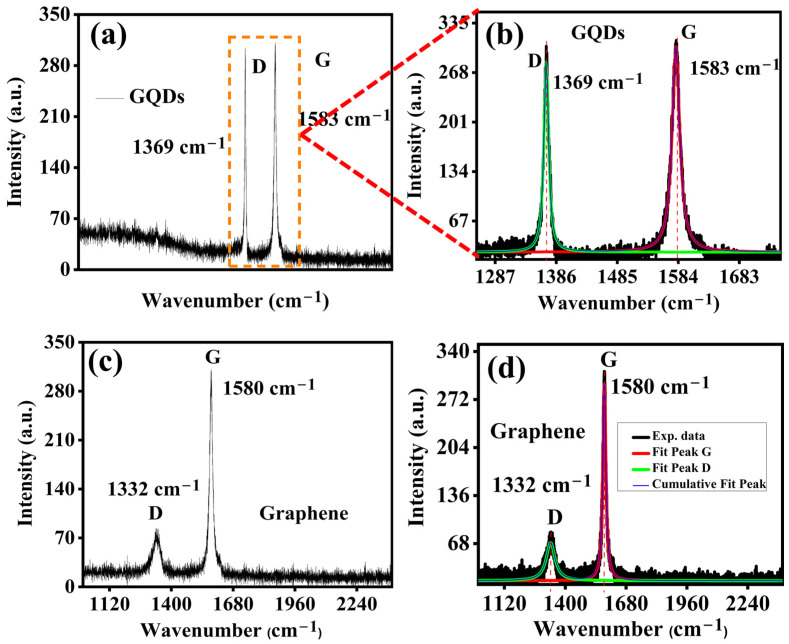
(**a**,**b**) Raman scattering D and G bands of GQDs; (**c**,**d**) graphene with Lorentz fitting.

A comparison of Raman spectra for graphene quantum dots and graphene is shown in Figure 3 for the excitation wavelength 532 nm. The deconvolution of the Raman spectrum is essential for understanding the disordered graphitic materials [28].

The typical graphite features include the D band (~1315–1370 cm^−1^) activated by the symmetry breaks on edges due to defects and the G band (~1400–1590 cm^−1^). The characteristic G band is due to the in-plane vibrations of sp^2^ carbon (E2g mode). Both samples materials investigated exhibited strong Raman signals with symmetrical bands which indicate structural homogeneity. The observed Raman shift in the D and G bands for GQDs points to the size-dependent phenomenon. As the D band behavior relates to the dispersive second-order double-resonance Raman scattering mode [29], so it can be attributed to the quantum confinement effect. The origin of the D mode involves optical excitation from the defect-related Raman modes. These excitations (which each consist of a defect or a D phonon) are followed by inter-valley scattering events of the electron or hole within the graphene Brillouin zone [29,30]. The size dependence of the D mode in GQDs is found to be similar to that in carbon nanotubes. Both the resonant phonon wave vector and the transition energy decreases as the diameter increases [31]. Thus, for nanotubes, the D mode is observed at lower frequencies as the diameter increases [31]. Following the same trend, this represents the lower size of quantum dots as responsible for the increase in the D mode frequency, as compared to the pristine graphene. An increased intensity of the Raman D band for GQDs can also be associated with the present special kind of disorder, named edge defect [32].

**Figure 4:**
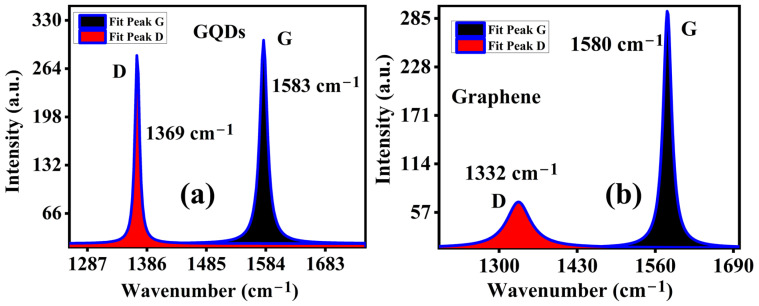
Lorentz fitting of peaks of D and G bands for (**a**) GQDs and (**b**) graphene. (The blue line indicates the cumulative peak area).

Figure 4 exhibits the Lorentz fitting of D and G bands presented in the selected area Raman spectra, which shows that the D bands of GQD and graphene have different linewidths (as the FWHM of the D band of GQD is only 8.78 cm^−1^, while the FWHM of the D band of graphene is 48.51 cm^−1^) [28]. The amplitude ratio of I_D_/I_G_ is less than 1 for both carbon material cases (for GQD 0.91 and graphene 0.24, respectively). The increase in the ratio of I_D_/I_G_ for GQDs suggests that the quantum dots of a lateral size L have a strong relationship with the Raman mode D [30]. Moreover, the inhomogeneity peak broadening in graphene may arise as a result of phonon confinement. It means that the non-zone-center phonon is also taking part in the Raman scattering process. This type of inhomogeneous peak broadening in graphene becomes significant when the size effect or the morphology modulation takes place. 

**Figure 5:**
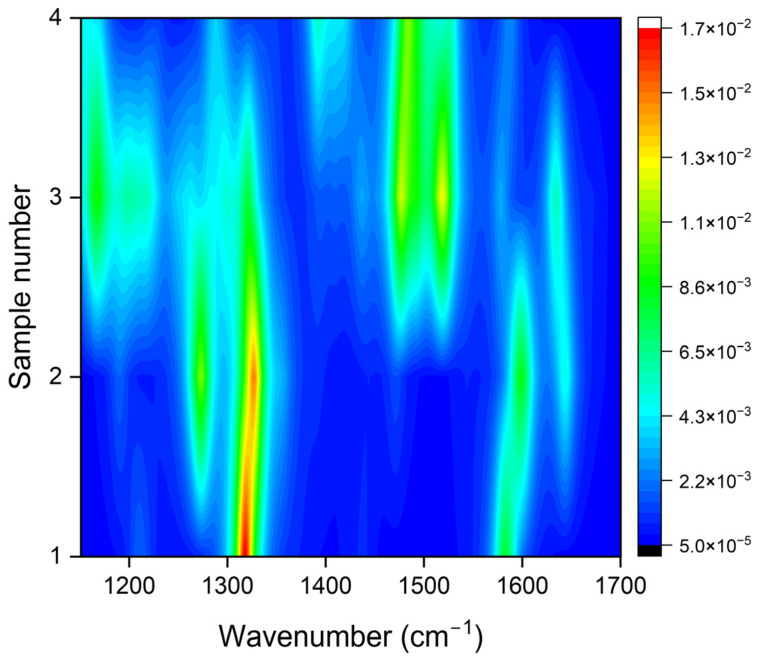
The 2D contour plot of theoretically simulated Raman spectra of GQDs with H-group, and OH-group attached outside (sample 1, and 2) and inside (sample 3, and 4) of the aromatic ring.

However, it is hard to predict the relationship between the origin of different Raman modes in GQDs, as it is highly controversial that researchers have claimed that different functional groups attached to the aromatic core of GQDs may tune the optical properties. It is also important to note that the position of attached functionalized group is also crucial to realize the significance of edge-effect or defect-mediated optoelectronics in GQDs. To investigate the role of these functional groups attached either outside or inside of the aromatic ring, we have theoretically calculated Raman spectra of GQDs (as shown in Figures S1 and S2). For this purpose, we used –H, and –OH functional groups to simplify the understanding and effect of these functional groups on the nature of Raman modes of GQDs. Interestingly, we observed that the frequency and intensity of D and G modes associated with GQDs were slightly different for each case. For instance, the D mode behaved prominently in the case where the functional groups were attached outside of the aromatic ring. However, the G mode became dominant when the functional groups were attached within the aromatic ring by creating defects (as shown in Figure 5). Figure 5 shows the 2D contour plot of the theoretically simulated selected range Raman spectra of GQDs (the source of the data is Figure S2) when the functional groups are attached outside (sample 1, and 2) and inside (sample 3, 4) of the aromatic ring structure. In addition, several other peaks also appear in the theoretical Raman spectra of GQDs (shown in Figure S2). However, the origin of these peaks is still unknown. We think that these peaks could be from the aromatic ring of the GQDs or from the bonding between C–H, and C–OH. After comparing with our experimental Raman spectra, we can conclude that the experimental Raman modes suggest there are functional groups attached at the edges of aromatic ring as well as within aromatic ring, behaving as defects. 

### 2.4. Photoluminescence Investigations

**Figure 6:**
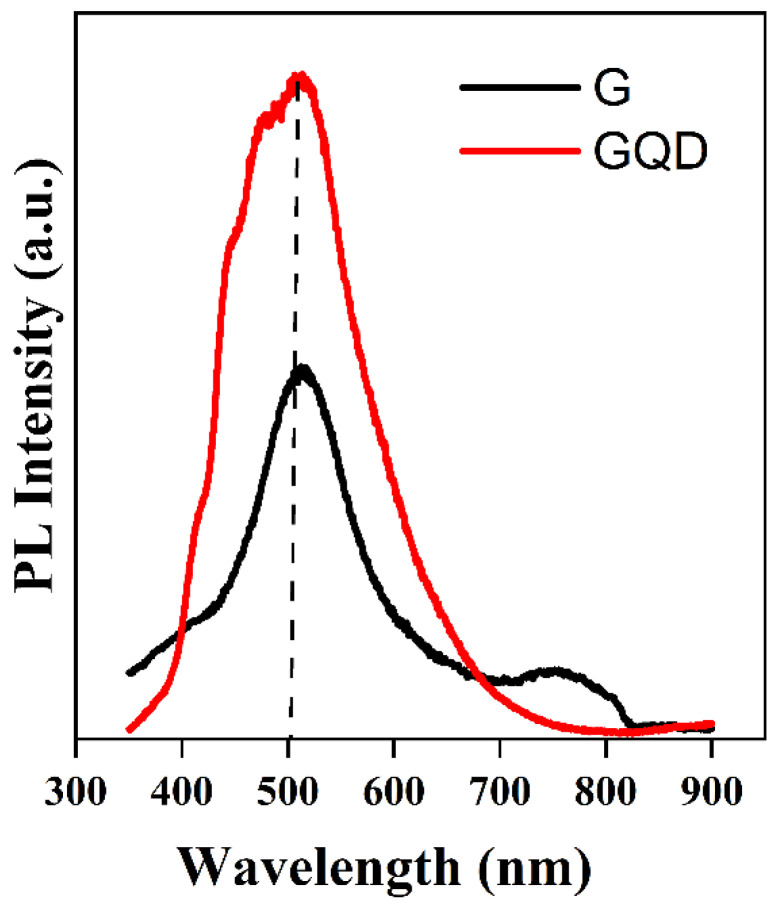
Room-temperature photoluminescence spectra of graphene and GQDs with a 325 nm excitation wavelength.

Figure 6 represents the room temperature photoluminescence signature of the graphene and GQD films deposited on SiO_2_ and glass substrate, respectively. As we know, a perfect graphene layer has no significant photoluminescence peak due to its zero-band gap. Naturally, unmodified graphene behaves like single-layer graphene, with semi-metal-like characteristics. Nevertheless, if an electric field is applied perpendicular to the graphene sheets it can create asymmetry between adjacent layers, which results in a tunable band gap and can be modulated by varying the electric field strength. Therefore, luminescence in graphene quantum dots and some specially engineered graphene small sheets has been observed and band gap has been introduced artificially [33,34]. 

The small size of quantum dots or graphene cut into small pieces may introduce sp^2^ islands in the π bands of graphene. As a result, some defects were also introduced in the structure. Various photoluminescence emission origins in graphene-related materials have been reported [33,35,36]. In these, the band gap transitions related to the conjugate π-bands and luminescence originating from complex origins such as defects are more well known. The luminescence properties of graphene and its fragments, like GQDs, arise from the radiative recombination of the e-h pair of sp^2^ carbon aromatic sites [37], correlated with FTIR bands. It is clearly shown in the figure that the near-band emission (NBE) peak intensity from GQDs is two times stronger as compared to graphene. 

**Figure 7:**
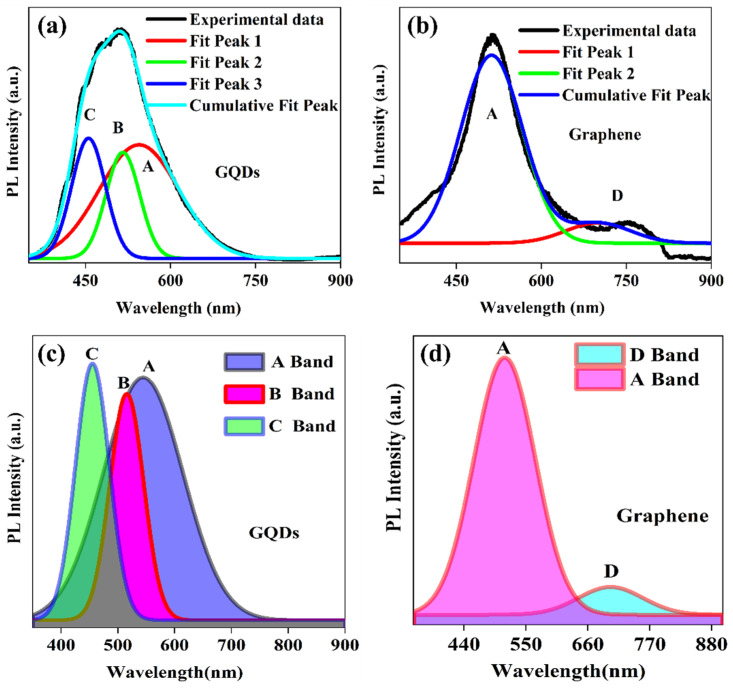
PL peak fitting of GQDs (**a**,**c**) and graphene (**b**,**d**) for the identification of different emission bands using Gaussian function.

Moreover, the peak in GQDs shows some other shoulder peaks which indicate that the NBE peak signal comprises some other feature peaks related to different interstate excitons, which mainly are considered as coming from different functional groups. For further elaboration, we performed Gaussian fitting to the PL spectra of graphene and GQDs, as shown in Figure 7. 

The GQDs show three peaks in the NBE region, named A, B, and C, of which A is associated with the e-h recombination and B and C could either be from the zigzag edge effect or from the functional group attached, respectively. However, graphene had only one A peak in NBE associated with the e-h recombination process with a defect peak (D) after 700 nm. This indicates that the enhanced luminescence in GQDs may result from the zigzag edge effect or from the functional group attached, which is presented only in quantum dots of small size. The FWHM of B and C peaks is also smaller than that of the e-h recombination process. It is also evident that more transitions contribute to these two phenomena and that transitions either from the functional group attached or from the edge states dominate in the case of GQDs.

**Figure 8:**
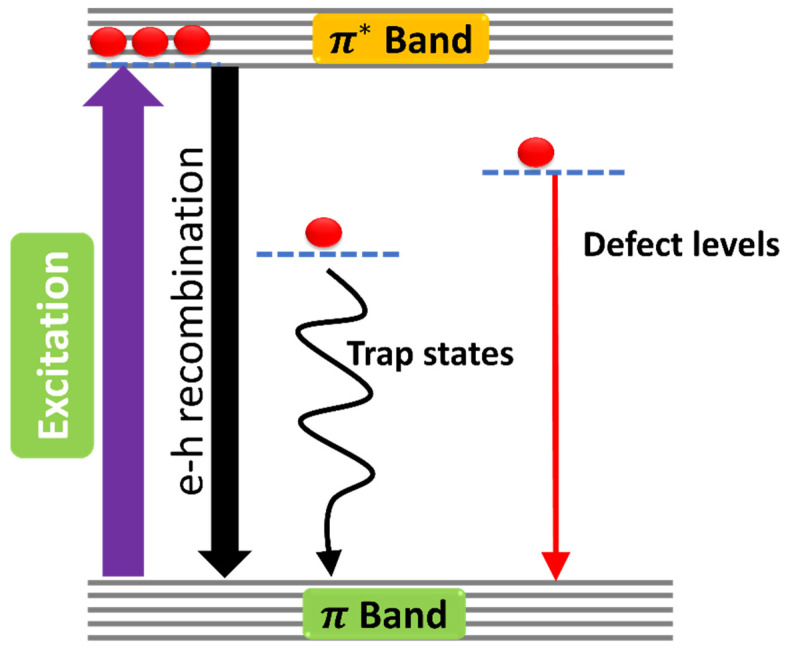
Schematic illustration of photoluminescence emission from different possible sublevels between π^*^ and π bands in graphene quantum dots.

To explain, the PL mechanisms of trap-mediated photoluminescence and defect-associated luminescence quenching in graphene are illustrated in Figure 8. The defects states may trap the excited electron from the upper state and give rise to red luminescence, which lies just after the visible spectrum.

Table 1 shows the fitting parameters of all fitted peaks, and one can see that the FWHM of the B band is smallest, which means that the trap-mediated luminescence peak is sharper as compared to others, and Figure 7c also shows the same. Moreover, the I_B_/I_A_ and I_C_/I_A_ are 0.93 and 1.05, respectively. The ratio of the intensity of the trap to the e-h recombination peak is almost equal to 1, which means that the trap-mediated luminescence strongly affects the near-band edge emission. 

The intense photoluminescence peak that arises from the B band in GQDs also predicts that with a smaller size, the quantum confinement effect strongly affects the Columbic interaction within e-h pairs, and the many-body system upholds the physical phenomenon at a smaller size [38,39]. The trap-mediated photoluminescence in GQDs is almost two times that in pristine graphene, which means the size has a significant effect on the activation of the trap states in low-dimension materials. 

**Figure 9:**
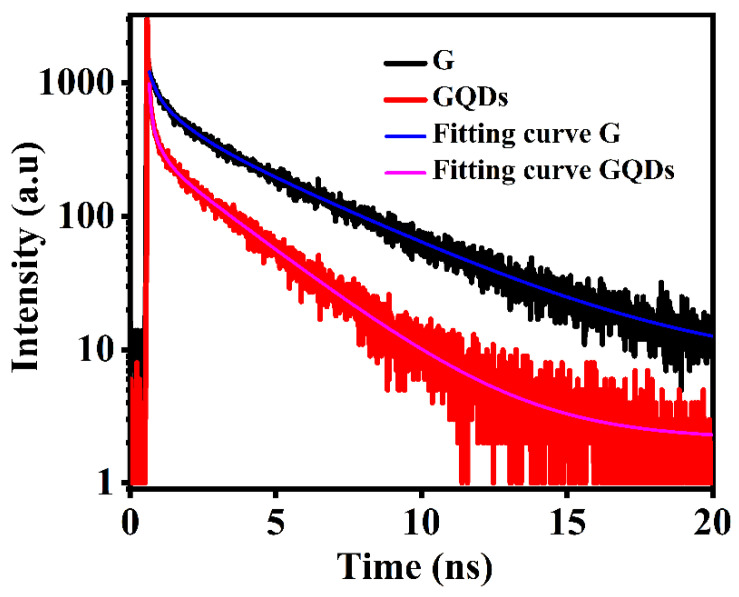
Time-resolved PL spectra of GQDs and graphene.

To further recognize the trap states’ role in photoluminescence and the radiative decay process, the lifetime (1/radiative decay rate) was measured using the bi-exponential decay function fitting (using the following Equation (1)) of experimental data obtained from TRPL (as shown in Figure 9) [40].

(1) It=C+∑i=1nAie−tτi

The average lifetime of charge carriers in graphene and GQDs has been calculated using the following equation.

(2) $$\tau =\frac{\sum_{i=1}^{n}A_{i}\tau_{i}^{2}}{A_{i}\tau_{i}}$$

Here, *C* is the fitting parameter (background offset) and $A_{i}$ denotes the amplitude of the component at *t* = 0; $\tau_{i}$ represents the single decay time of carriers. According to the above equations, the average lifetime $\tau_{ave}$ of carriers calculated is 4.87 ns and 2.95 ns for graphene and GQDs, respectively. 

Our results are consistent with previously reported results in which it was suggested that the large photoluminescence enhancement from GQDs may arises from edge states or from the size effect [40,41,42]. As we know that the trap states are not so stable, that may be why the carrier decay curve in the GQDs shows quicker radiative decay as compared to the graphene. Moreover, the photogenerated electrons and holes in small-sized GQDs may take time to reach the trap states before they recombine and then relax their energies to the shallow localized states. Considering all this, the near-band edge luminescence enhancement can come from the activation of trap states or from the functionalized group attached to graphene quantum dots.

### 2.5. DFT Calculations

**Figure 10:**
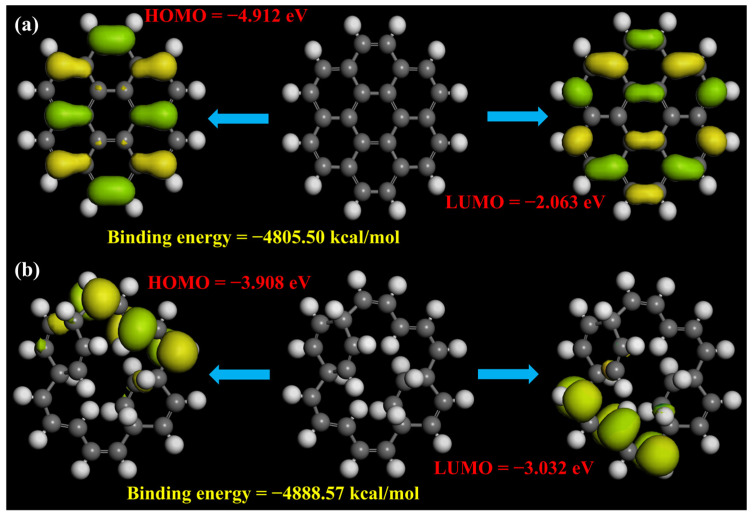
DFT calculation using the DMol^3^ code for HOMO, LUMO and binding energies of (**a**) H-group attached outside, and (**b**) H-group attached inside of the aromatic ring of the 7-ring structure of GQDs.

**Figure 11:**
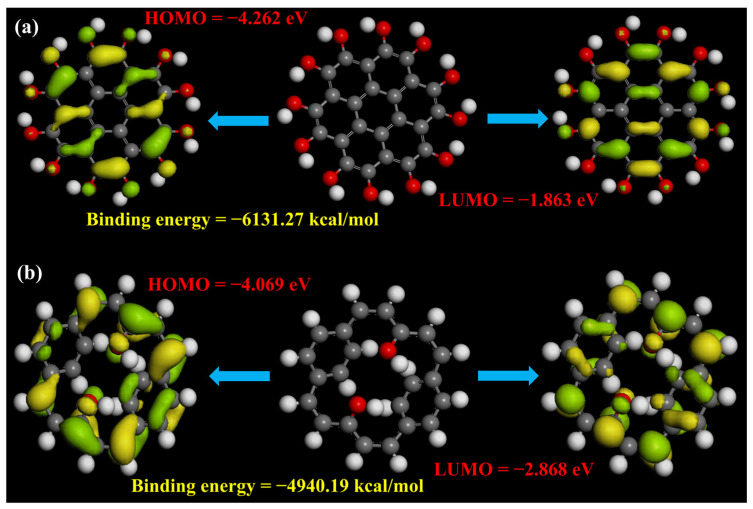
DFT-calculated HOMO, LUMO and binding energies of (**a**) OH-group attached outside, and (**b**) OH-group attached inside of the aromatic ring of the 7-ring structure of GQDs.

Since the photoluminescence signature in GQDs is a complex and more controversial topic in this field, to figure out the origin of the enhanced photoluminescence in GQDs, a density functional theory (DFT)-based simulation was developed to systematically study the effect of different functional groups and their position with respect to the aromatic ring (the details of the theoretical simulation have been provided in the Supplementary Information, and DFT-calculated binding energies, HOMO and LUMO values, zero-point vibrational energies, and the dipole magnitude of the –H, and –OH groups attached inside and outside the aromatic ring of GQDs with a seven-ring structure are summarized in Table S1). In short, we calculated the electronic structure of GQDs with –H and –OH functional groups attached at the edges and within the aromatic ring for the simplest seven-ring GQDs structure. This approach was adopted due to the experimental FTIR results, in which the –OH peak contains the highest intensity for the as-synthesized GQDs. Previous reports indicate that the main reactions take place at the edges of the GQDs [7,32]. Therefore, we arranged and compared the simulation results of functionalized groups attached outside and inside of the aromatic ring. Here, it is worth mentioning that for the purposes of geometrical optimization and hydrogen adjustment, we also attached hydrogen at the edges for the calculation of the functional group inside the aromatic ring model. Figure 10 represent the highest occupied molecular orbital (HOMO) and lowest unoccupied molecular orbitals (LUMO) of the GQDs with the –H group attached outside and inside of the aromatic ring structure. It can be seen that in the case of the H-group attached outside, the energy states arise from self-orbitals of functional groups as well as from the long-range π-hybridization [11,22]. However, in the case of the –H group attached inside the aromatic ring, only the energy states from self-orbitals of the functional groups dominated. We believe that these self-orbitals of the functional groups make a major contribution towards the carrier dynamics of the GQDs, as the charge potential graph (shown in Figure S3) and previous reports indicate that the high charge density appeared near these self-orbitals of functional groups and was comparable with our theoretical density of states calculation, which suggests that high electron density has been reported in the p-orbital (as shown in Figure S4) [21,22]. In addition, the HOMO and LUMO values of the H-group attached outside and inside of the aromatic ring indicate a huge difference, suggesting that the bandgap shrinks when the H-group is attached inside of the aromatic ring, which indicates that the size of the GQDs expands as their emission shifts in the higher wavelength range of the visible spectrum (as shown in Figure S5). Furthermore, a similar trend has been shown in the case of the OH-group, where the bandgap value decreases from 2.399 eV to 1.201 eV. In other words, this indicates that the position of the functional group modulates the emission wavelength highly in the case of low-dimensional systems such as GQDs. Figure 11 represents the HOMO, LUMO structure of seven-ring GQDs with the –OH group attached outside (Figure 11a) and inside (Figure 11b) of the aromatic ring. It has been observed that the HOMO levels of these functionalized GQD structures are naturally hybridized C–C π orbitals (residing at the edges of the aromatic ring), from disordered structures and from the functional group attached (which has much less influence, though, in modulating the HOMO levels in the present case, as they have very low isosurface intensity). Furthermore, in the case of the –OH group attached outside the aromatic ring, the long-range π hybridization dominates over the self-orbitals of functional groups as the density of π hybridization is high, which suggests that the emission from this model can be dictated by the long-range π hybridization which arises from the aromatic ring of GQDs [18,22]. On the other hand, self-orbitals of functional groups have higher density when the –OH group is attached within the aromatic ring structure (as shown in Figure 11b), indicating that long-range π hybridization weakens in this model and the emission from this type of structure is mainly attributed to the edge effects or the self-orbitals of functional groups.

Additionally, from theoretical and experimental bandgap analysis, it can be seen that the OH-functional group attached outside of the aromatic ring perfectly matched with our experimental results, as the experimental bandgap (2.407 eV) was comparable to the theoretical simulated bandgap (2.399 eV) of the OH-group attached outside of the aromatic ring (as shown in Figure S5). In a similar argument, several previous reports also pointed out the fact that oxygen-containing functional groups are responsible for tailoring the emission wavelength in GQDs [11,22,43]. The comparative analysis of the HOMO-LUMO bandgap of different functionalized GQDs has been summarized in Table S2. In short, oxygen-containing functionalized GQDs with a functional group attached outside of the aromatic ring display a HOMO-LUMO bandgap similar to the experimental bandgap. Furthermore, the position of functional group attachment to the aromatic ring is also significant to tailor the HOMO-LUMO energy level of GQDs.

## 3. Materials and Methods

As-prepared graphene quantum dots (Nanjing XFNANO Materials Tech Co., Ltd., Nanjing, China) with a concentration of 1 mg/mL were dispersed in the ethanol solution (1:2 ratio). The prepared GQDs solution was sonicated for 40 min. After that, the GQDs were deposited onto the 0.5 by 0.5 mm glass slide substrates, the same as those used in our previous work [44], with spin coating in the ambient environment at 500 rpm. For comparison, we also used bilayer graphene sheet (Nanjing XFNANO Materials Tech Co., Ltd., Nanjing, China) deposited on 7 × 7 cm SiO_2_ substrate. Prior to the deposition of GQDs, all the substrates were ultrasonically cleaned for 20 min each, using acetone, ethanol, and deionized water, respectively. The cleaned substrates were then dried with 99.999% Nitrogen to remove the dust and organic impurities. After the deposition, the samples were placed under a 100 W Xenon lamp for 30 min to allow the film to dry. 

A field emission electron microscope (FESEM) model JSM 6500F (JEOL, Tokyo, Japan) was used to characterize the surface morphology of the deposited graphene films. 

A transmission electron microscope (TEM) model H-9500 (Hitachi, Tokyo, Japan) was used for size detection of the GQDs. 

Fourier-transform infrared spectroscopy (FTIR) spectra for samples were recorded by a Bruker Alpha-T spectrometer (Bruker Optik GmbH, Ettlingen, Germany).

Raman measurements were taken using laser excitation wavelengths of 532 nm. Laser power was kept at 1 mW to prevent the heating or damaging of the samples. Spectra were obtained using 2 min integration times and were collected from several areas of samples to assess the homogeneity of these structures. 

A PL spectrometer with a He-Cd laser with a wavelength of 325 nm at room temperature was used to measure the photoluminescence spectra. The photoluminescence spectra were measured using the same configuration as used in our previous work [45]. 

The time-resolved photoluminescence (TRPL) results were attained using a time-correlated single-photon counting system in right angle sample geometry with a 379 nm picosecond laser with a pulse repetition rate of 2 MHz (Edinburgh Instruments, Livingston, UK, EPL375). The theoretical simulation of GQDs was carried out using the DMol^3^ module of BIOVIA Materials Studio 2020 (version 20.1.0.2728). The geometry optimization was performed using GGA pseudopotential.

## 4. Conclusions

In summary, the role of a functional group attached at the edges as well as within the aromatic ring of GQDs to tailor the luminescence signature has been analyzed in graphene and graphene quantum dots. The strong trap-mediated photoluminescence signatures in GQDs at room temperature have been found to arbitrate the luminescence efficiency by two-fold from graphene, which indicates the perspective of GQDs as a good candidate for the fabrication of luminescence devices used in bio-imaging and optoelectronics. In addition, the theoretical and experimental results reveal that the oxygen-containing functional groups attached outside of the aromatic rings are the main source of luminescence from GQDs, as they contain long-range π-hybridization as well as self-orbitals from functional groups which contributed to the enhancement of the emission wavelength. Furthermore, our findings show no evidence of luminescence tailoring of GQDs by functional groups attached within the aromatic ring. 

## Figures and Tables

**Table 1:** The fitting parameters of different peaks from the near-band-edge emission band and defect band in PL spectra of GQDs and graphene.

GQDs				Graphene	
Bands	A	B	C	A	D
Peak Position (nm)	544.52 ± 1.22	515.79 ± 0.90	455.34 ± 0.82	513.18 ± 0.16	752.62 ± 1.04
Peak Intensity (a.u.)	1.105 × 10^6^	436,141.01	496,059.13	1.293 × 10^6^	78,384.74
FWHM (nm)	165.41 ± 1.42	69.96 ± 1.30	70.18 ± 1.25	121.61 ± 0.71	65.28 ± 4.08

## Data Availability

Data is contained within the article or Supplementary Materials.

## Supplementary Materials

The following supporting information can be downloaded at: https://www.mdpi.com/article/10.3390/molecules29122790/s1, Figure S1: Theoretically simulated survey Raman spectra of –H and –OH group attached inside and outside of the aromatic ring in GQDs, with the simulation performed at 300 K with incident wavelength of 532 nm; Figure S2: Theoretically simulated selected range (1150–1700 cm^−1^) Raman spectra of –H and –OH groups attached inside and outside of the aromatic ring in GQDs, with the simulation performed at 300 K with incident wavelength of 532 nm; Figure S3: The electrostatic potential mapping of (a,c) H-group attached outside and inside and (b,d) OH-group attached outside and inside of the aromatic ring of the 7-ring GQD structure; Figure S4: The DFT-calculated density of states of (a,c) H-group attached outside and inside and (b,d) OH-group attached outside and inside of the aromatic ring of the 7-ring GQD structure; Figure S5: HOMO and LUMO energy levels for H-group and OH-group attached outside and inside of the aromatic ring, and their respective bandgap energy. Sample numbers correspond to the respective case, as mentioned in Table S1; Table S1: DFT-calculated binding energies, HOMO, LUMO values, zero-point vibrational energies, and dipole magnitude of –H and –OH groups attached inside and outside of the aromatic ring of GQDs with a 7-ring structure. Table S2: Comparative analysis of GQDs with different functional groups or dopant attached at different positions and their respective HOMO-LUMO energy gap. References [18,46,47,48,49,50,51,52,53] are cited in the supplementary materials.
