# Supplementary material for: Role of Functional Groups in Tuning Luminescence Signature of Solution-Processed Graphene Quantum Dots: Experimental and Theoretical Insights
Source: Molecules. 2024 Jun 12;29(12):2790. doi: 10.3390/molecules29122790 (PMC11206256; doi:10.3390/molecules29122790)
Supplement: Supplementary file 1 [file molecules-29-02790-s001.zip › molecules-3029732-supplementary.pdf]

# Supplementary information

# Role of Functional Groups in Tunning Luminescence Signature of Solution-Processed Graphene Quantum Dots: Experimental and Theoretical Insights

**Zhicheng Ke[1], Muhammad Azam[2], Shujat Ali[3,*], Muhammad Zubair[4], Yu Cao[1], Abbas Ahmad Khan[5,*], Ali Hassan[1,6*], Wei Xue[1,7]**

1. China International Science and Technology Cooperation base for Laser Processing Robotics, Wenzhou University, Wenzhou 325035, P.R. China alirao@wzu.edu.cn (A.H), 22461440046@stu.wzu.edu.cn (Z.K), yucao@wzu.edu.cn (Y.C)
2. National Key Laboratory of Electronic Films and Integrated Devices, School of Integrated Circuit Science and Engineering, University of Electronic Science and Technology of China, 610054 Chengdu, P.R. China mazam796@uestc.edu.cn (M.A)
3. College of Electrical and Electronic Engineering, Wenzhou University, Wenzhou, 325035, P.R. China 20202024@wzu.edu.cn (S.A)
4. Forschungszentrum Jülich GmbH, Institute of Energy and Climate Research; Materials Synthesis and Processing (IEK-1), 52428 Jülich, Germany m.zubair@fz-juelich.de (M.Z)
5. IMDEA-Nanociencia, Campus de Cantoblanco, 28049 Madrid, Spain abbas.khan@imdea.org (A.A.K)
6. Zhejiang provincial Key laboratory of Laser Processing Robotics, College of Mechanical and Electrical Engineering, Wenzhou University, Wenzhou 325035, P.R. China alirao@wzu.edu.cn (A.H)
7. Oujiang Laboratory (Zhejiang Lab for Regenerative Medicine, Vision and Brain Health), Wenzhou University, Wenzhou 325000, China xm@wzu.edu.cn (W.X)

* Correspondence: alirao@wzu.edu.cn (A.H.), abbas.khan@imdea.org (A.A.K) , 20202024@wzu.edu.cn (S.A)

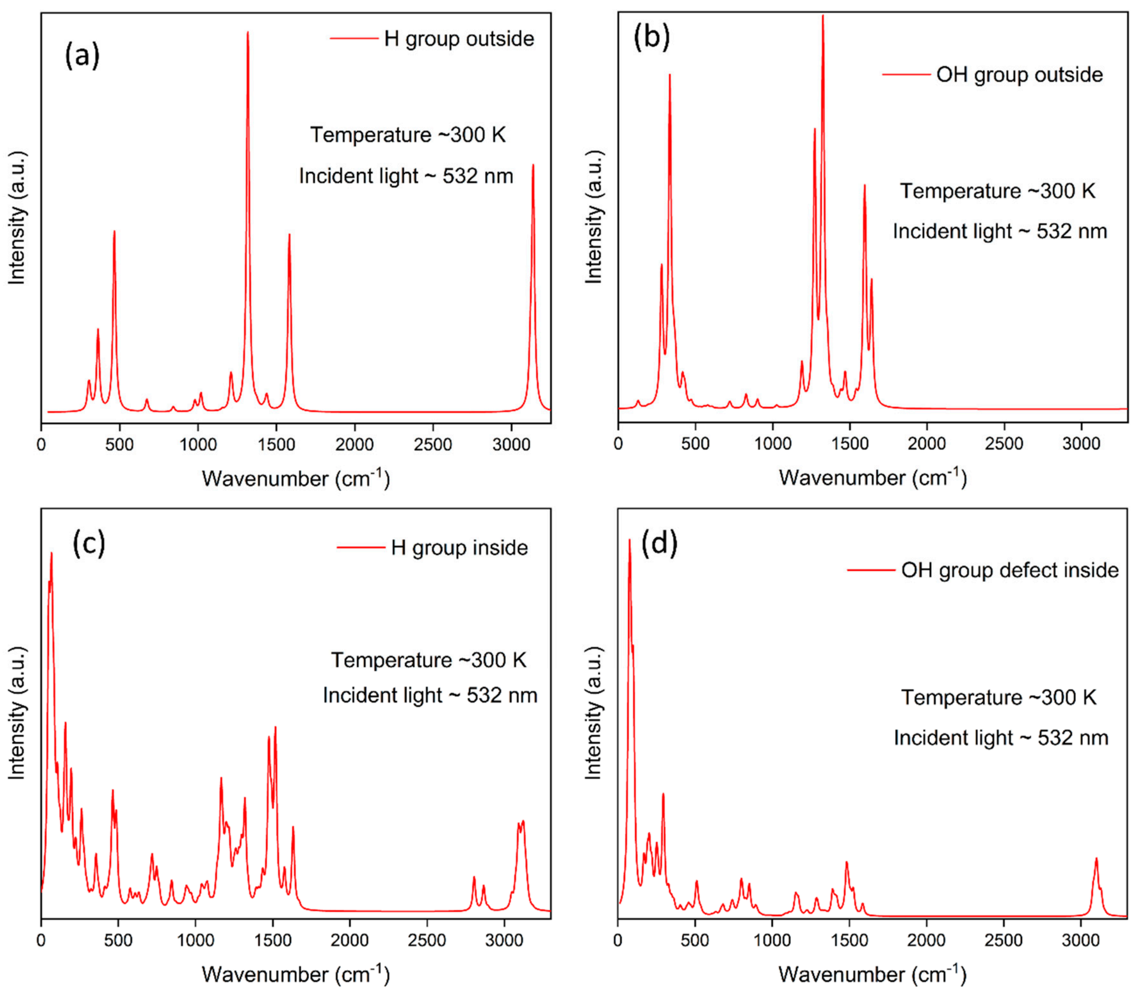


**Figure S1:** Theoretically simulated survey Raman spectra of -H, and -OH group attached inside and outside of the aromatic ring in GQDs, the simulation performed at 300 K with incident wavelength of 532 nm.

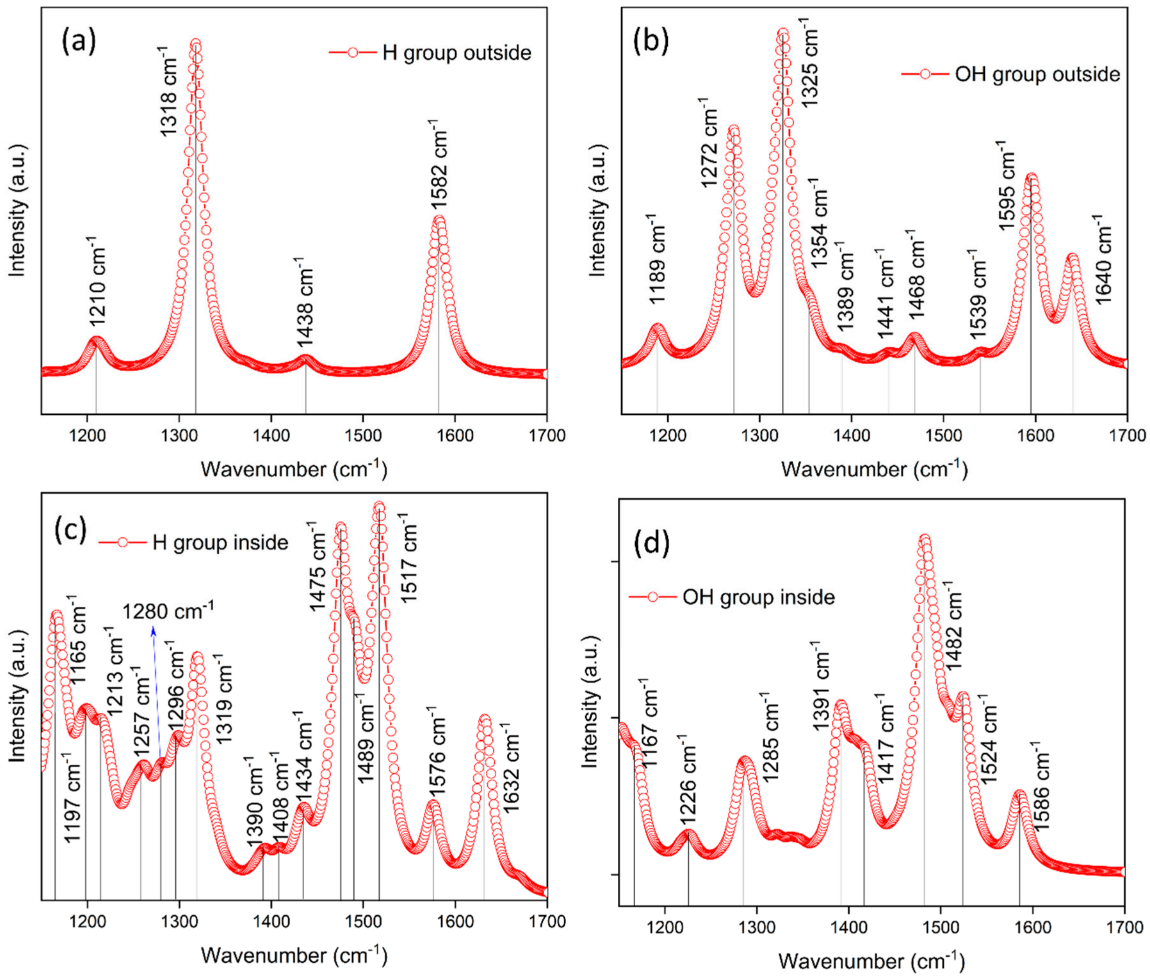


**Figure S2:** Theoretically simulated selected range (1150-1700 cm$^{-1}$) Raman spectra of -H, and -OH group attached inside and outside of the aromatic ring in GQDs, the simulation performed at 300 K with incident wavelength of 532 nm.

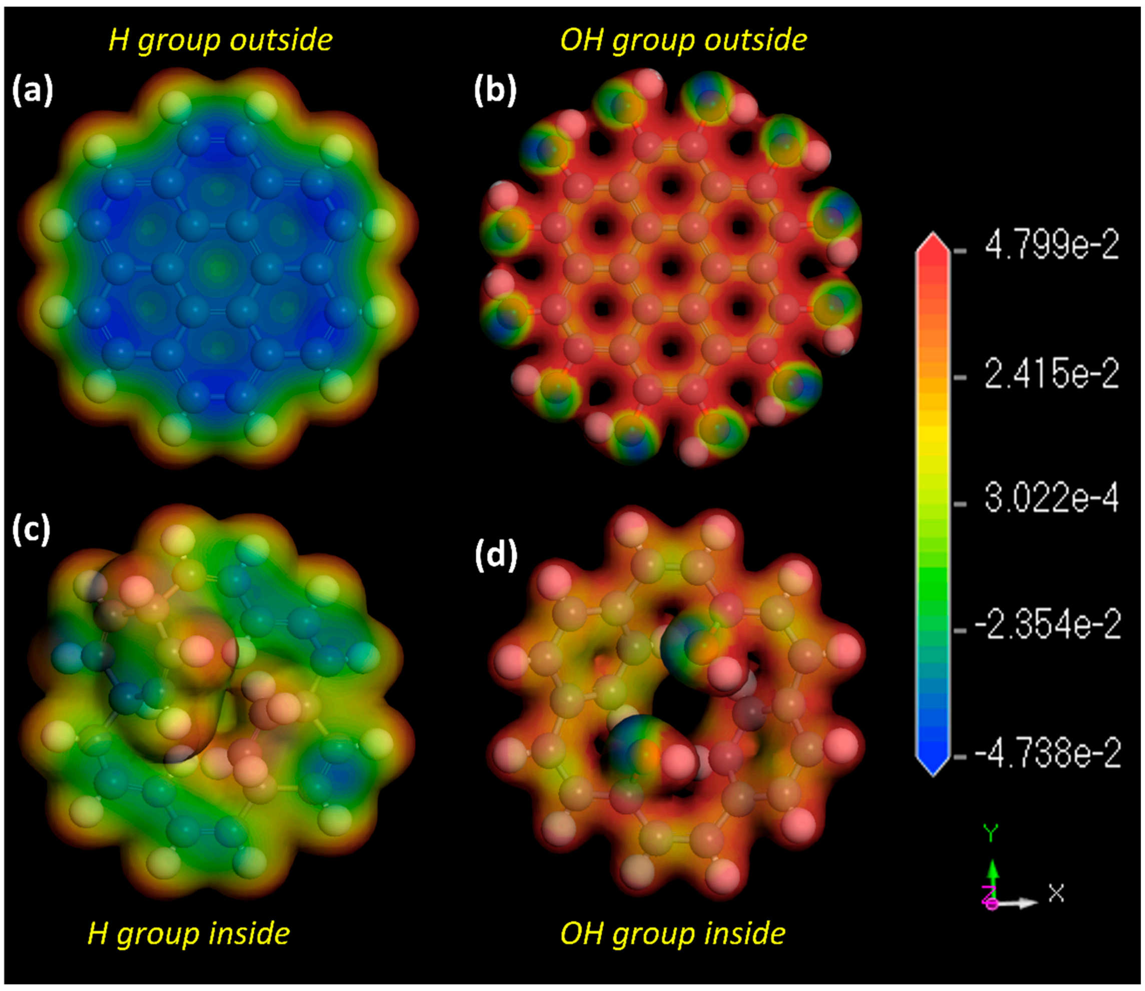


**Figure S3:** The electrostatic potential mapping of (a, c) H-group attached outside and inside, and (b, d) OH-group attached outside and inside of the aromatic ring of 7-ring GQD structure.

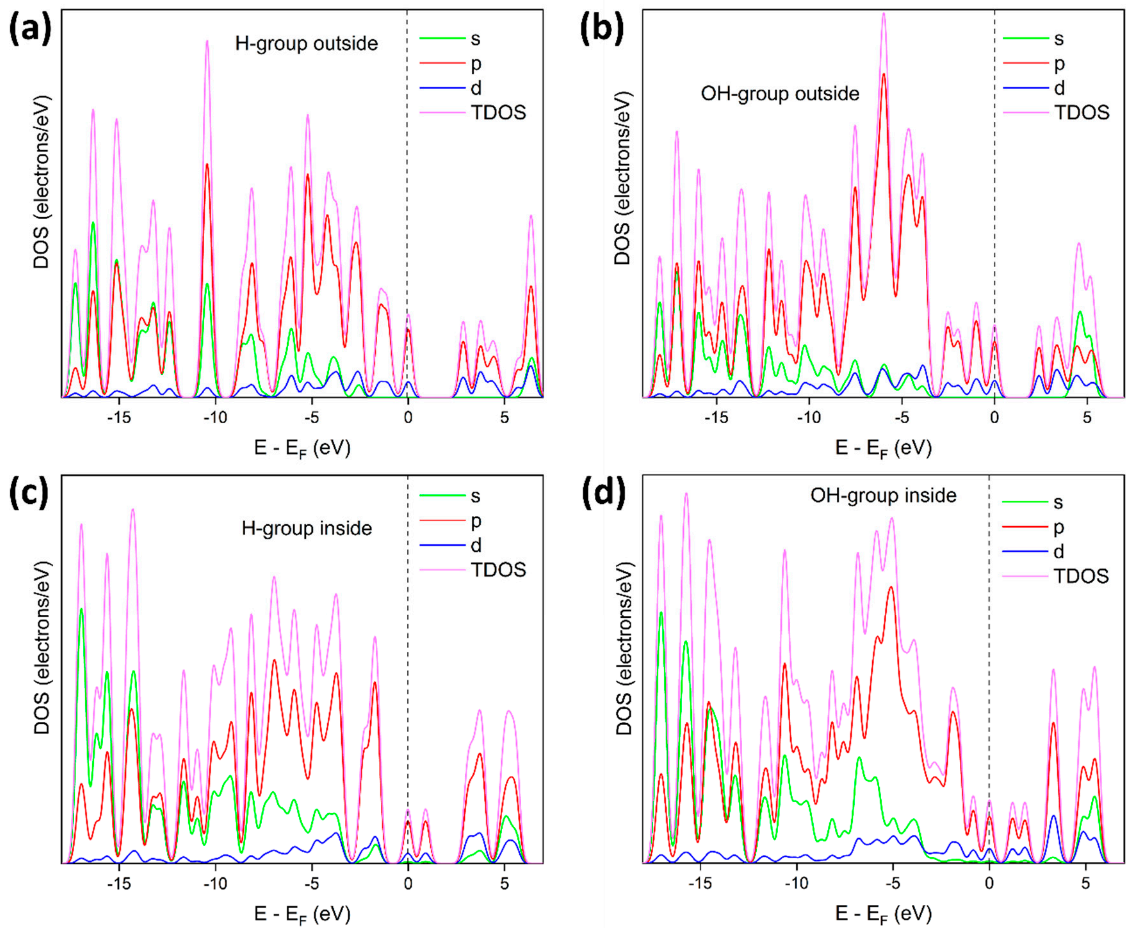


**Figure S4:** The DFT calculated density of states of (a, c) H-group attached outside and inside, and (b, d) OH-group attached outside and inside of the aromatic ring of 7-ring GQD structure.

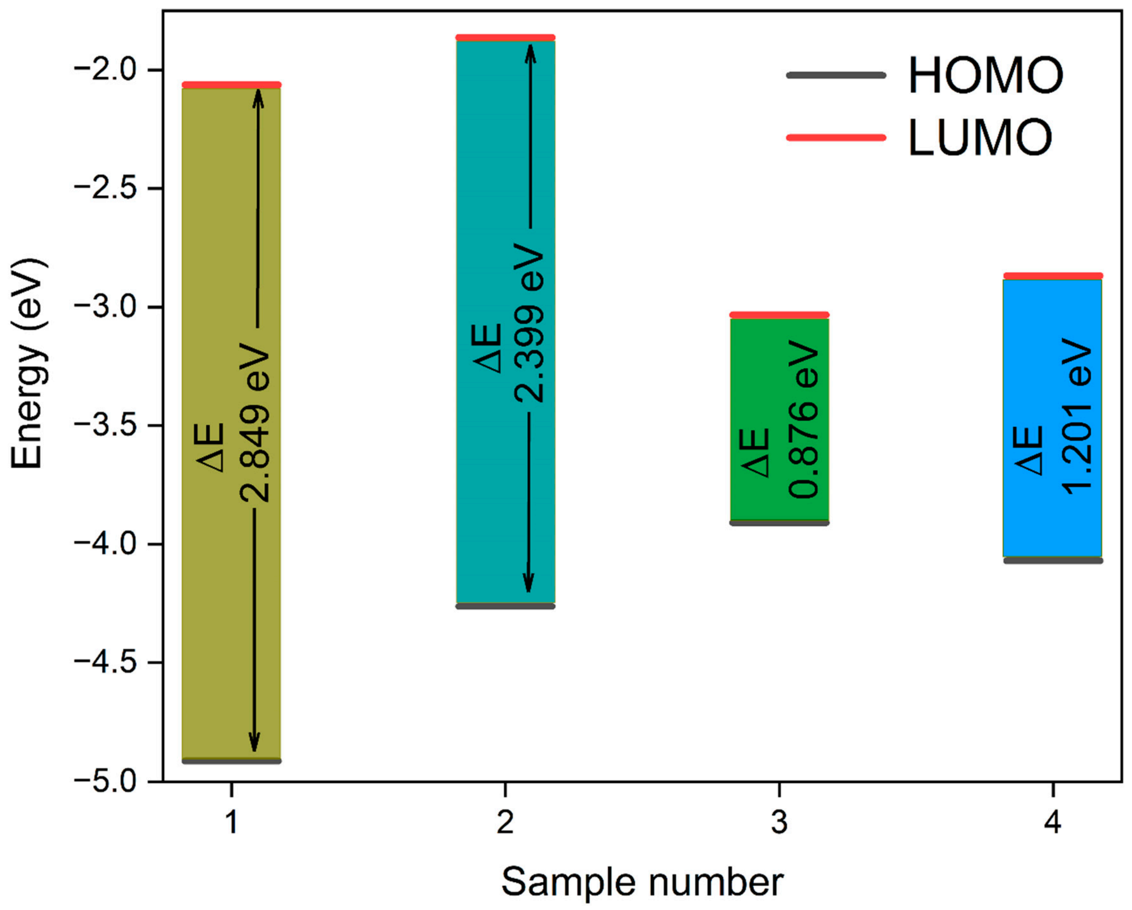


**Figure S5:** HOMO and LUMO energy levels for H-group and OH-group attached outside and inside of the aromatic ring, and their respective bandgap energy. Sample numbers correspond to the respective case as mentioned in Table.S1.

**Table.S1:** DFT calculated binding energies, HOMO, LUMO values, zero-point vibrational energies, and dipole magnitude of -H, and -OH group attached inside and outside of the aromatic ring of GQDs having 7-ring structure.

| Sample no. | Functional group and its position | Binding Energy (kcal/mol) | HOMO (eV) | LUMO (eV) | Zero-point vibrational energy (kcal/mol) | Dipole magnitude (debye) |
|---|---|---|---|---|---|---|
| 1 | H group outside | -4805.50 | -4.912 | -2.063 | 171.601 | 0.0012 |
| 2 | OH group outside | -6131.27 | -4.262 | -1.863 | 201.967 | 0.0337 |
| 3 | H group inside | -4888.57 | -3.908 | -3.032 | 225.646 | 0.2800 |
| 4 | OH group inside | -4940.19 | -4.069 | -2.868 | 205.017 | 0.9324 |

**Table.S2:** Comparative analysis of GQDs having different functional groups or dopant attached at different positions and their respective HOMO-LUMO energy gap.

| **GQDs (ring size/structure)** | **Functional group (Inside/Outside)** | **HOMO-LUMO gap (eV)** | **Ref.** |
|---|---|---|---|
| Zigzag ($C_{42}H_{18}$) | COOH (outside) | 2.762 | [1] |
| Armchair ($C_{54}H_{18}$) | COOH (outside) | 3.542 | |
| 48-ring $C_{132}$-EF (edge functionalized) | OH (outside) | 2.34 | [2] |
| | $OCH_3$ (outside) | 2.31 | |
| | COOH (outside) | 2.32 | |
| | CHO (outside) | 2.30 | |
| | COC (outside) | 2.15 | |
| 19-ring ($C_{54}H_{18}$) | H group (outside) | 2.82 | [3] |
| 13 ring ($C_{42}H_{18}$ ) | H group (outside) | 3.59 | [4] |

| | | | |
|---|---|---|---|
| 7-ring ($C_{24}H_{12}$) | H group (outside) | 2.83 | [5] |
| | OH group (outside) | 2.37 | |
| 7-ring $C_{24}H_{12}$ | H group (outside) | 2.849 | **This work** |
| 7-ring $C_{24}H_{12}O_{12}$ | OH group (outside) | 2.399 | |
| 7-ring $C_{22}H_{12}$ | H group (Inside) | 0.876 | |
| 7-ring $C_{22}H_{18}O_2$ | OH group (Inside) | 1.201 | |

**Supplementary Note:**

The theoretical simulation study of GQDs with different functional groups attached outside and inside aromatic ring structure has been carried out using BIOVIA Materials Studio version 2020 (version 20.1.0.2728 ) using DMol$^3$ module which uses density functional theory (DFT) to simulate chemical processes theoretically and predict several electronic, structural, and optical properties both rapidly and accurately [2, 6, 7]. The exchange-correlation energy used in DMol$^3$ is given by Eq. (S1),

$$E_{xc}[\rho] \cong \int \rho(r)\, \varepsilon_{xc}[\rho(r)]dr \qquad \text{(S1)}$$

Here $\varepsilon_{xc}[\rho]$ is the exchange-correlation energy which is calculated for one particle in a uniform electronic gas. We performed geometry optimization using GGA function with additional nonlocalized Becke exchange plus Lee-Yang-Parr [BLYP] correlation [8, 9]. The convergence tolerance quality was adjusted at 1.0 e$^{-5}$ Ha with a maximum force of 0.002 Ha/Å and maximum iterations of 50. For numerical basis sets, the matric elements needed to solve the self-consistent field (SCF) equations and to calculate total energy are given by the following DFT equations,

$$H_{\mu\nu} = \left\langle \chi_\mu(r_1) \middle| -\frac{\nabla^2}{2} + V_N + V_e + \mu_{xc}[\rho(r_1)] \middle| \chi_v(r_1) \right\rangle \quad \text{(S2)}$$

And,

$$S_{\mu\nu} = \langle \chi_\mu(r_1) | \chi_\nu(r_1) \rangle \quad \text{(S3)}$$

The numerical orbitals used in $DMol^3$ for the basis functions correspond to atomic orbitals (AO). The atomic basis sets are confined to a specific cutoff value which should be appropriate for a particular simulation quality level of $DMol^3$ calculations. This feature leads to much faster and more accurate calculations. In the present case, we used “Global” orbital cutoff scheme with “Fine” quality and with a cutoff value of 3.7 Å. Furthermore, the core treatment was set for “All Electron” from the “Electronic” tab with double numerical plus polarization (DNP) basis set function having a basis file of 3.5. In addition, we used solvation method in our calculations to make the simulation environment similar as the experimental synthesis of GQDs. The Solvent “Ethanol” was selected from the COSMO tab with having a dielectric constant of 24.3. Furthermore, the multipolar expansion was set to “Hexadecapole” with a charge and spin density mixing of 0.2 and 0.5, respectively. After the geometrical optimization step was completed, we performed properties calculation task on the optimized molecular structure to analysis the structural, electronic and vibrational properties. The theoretical Raman intensity was calculated using Lorentzian smearing with 20 $cm^{-1}$, and incident light of 532 nm at room temperature. All the values of HOMO, LUMO, Zero-point vibrational, Binding energies, and dipole moments for each set have been noted from the job completion 3D atomistic file.

**References:**

[1] H. Abdelsalam, H. Elhaes, M.A. Ibrahim, First principles study of edge carboxylated graphene quantum dots, Physica B: Condensed Matter, 537 (2018) 77-86.
[2] J. Feng, H. Dong, L. Yu, L. Dong, The optical and electronic properties of graphene quantum dots with oxygen-containing groups: a density functional theory study, Journal of Materials Chemistry C, 5 (2017) 5984-5993.
[3] J. Feng, Q. Guo, N. Song, H. Liu, H. Dong, Y. Chen, L. Yu, L. Dong, Density functional theory study on optical and electronic properties of co-doped graphene quantum dots based on different nitrogen doping patterns, Diamond and Related Materials, 113 (2021) 108264.
[4] J. Feng, H. Dong, B. Pang, F. Shao, C. Zhang, L. Yu, L. Dong, Theoretical study on the optical and electronic properties of graphene quantum dots doped with heteroatoms, Physical Chemistry Chemical Physics, 20 (2018) 15244-15252.
[5] M. Ojeda-Martínez, A.N. Pérez Martínez, J. El Hamdaoui, M. Courel Piedrahita, E.M. Feddi, S.P. Thirumuruganandham, M.L. Ojeda Martínez, J.L. Cuevas Figueroa, C. Velásquez Ordoñez, D.J. Mowbray, Tuning the energy gap of graphene quantum dots functionalized by OH and COOH radicals: First principle study, Materials Chemistry and Physics, 311 (2024) 128543.
[6] A.A. Abozeed, O. Younis, A.F. Al-Hossainy, N.A. El-Mawla, M. Sayed, A. M. Kamal El-dean, M.S. Tolba, Combined experimental and TD-DFT/DMOl3 investigations, optical properties, and photoluminescence behavior of a thiazolopyrimidine derivative, Scientific Reports, 12 (2022) 15674.
[7] A. Kumar, M.I. Sayyed, D. Punina, E. Naranjo, E. Jácome, M.K. Abdulameer, H.J. Albazoni, Z. Shariatinia, Graphene quantum dots (GQD) and edge-functionalized GQDs as hole transport materials in perovskite solar cells for producing renewable energy: a DFT and TD-DFT study, RSC Advances, 13 (2023) 29163-29173.
[8] A.D. Becke, A multicenter numerical integration scheme for polyatomic molecules, The Journal of Chemical Physics, 88 (1988) 2547-2553.
[9] C. Lee, W. Yang, R.G. Parr, Development of the Colle-Salvetti correlation-energy formula into a functional of the electron density, Physical Review B, 37 (1988) 785-789.
